# Critical but commonly neglected factors that affect contrast medium administration in CT

**DOI:** 10.1186/s13244-024-01750-4

**Published:** 2024-08-28

**Authors:** Michael C. McDermott, Joachim E. Wildberger, Kyongtae T. Bae

**Affiliations:** 1 Department of Radiology & Nuclear Medicine, Maastricht University Medical Center + , Maastricht, The Netherlands; 2 CARIM School for Cardiovascular Diseases, Maastricht University, Maastricht, The Netherlands; 3 Bayer AG, Berlin, Germany; 4 Department of Diagnostic Radiology, Li Ka Shing Faculty of Medicine, The University of Hong Kong, Hong Kong, Hong Kong

**Keywords:** Contrast media, Injections, Intravenous, Injection systems, Computed tomography

## Abstract

**Objective:**

Past decades of research into contrast media injections and optimization thereof in radiology clinics have focused on scan acquisition parameters, patient-related factors, and contrast injection protocol variables. In this review, evidence is provided that a fourth bucket of crucial variables has been missed which account for previously unexplained phenomena and higher-than-expected variability in data. We propose how these critical factors should be considered and implemented in the contrast-medium administration protocols to optimize contrast enhancement.

**Methods:**

This article leverages a combination of methodologies for uncovering and quantifying confounding variables associated with or affecting the contrast-medium injection. Engineering benchtop equipment such as Coriolis flow meters, pressure transducers, and volumetric measurement devices are combined with small, targeted systematic evaluations querying operators, equipment, and the physics and fluid dynamics that make a seemingly simple task of injecting fluid into a patient a complex and non-linear endeavor.

**Results:**

Evidence is presented around seven key factors affecting the contrast-medium injection including a new way of selecting optimal IV catheters, degraded performance from longer tubing sets, variability associated with the mechanical injection system technology, common operator errors, fluids exchanging places stealthily based on gravity and density, wasted contrast media and inefficient saline flushes, as well as variability in the injected flow rate vs. theoretical expectations.

**Conclusion:**

There remain several critical, but not commonly known, sources of error associated with contrast-medium injections. Elimination of these hidden sources of error where possible can bring immediate benefits and help to drive standardized and optimized contrast-media injections.

**Critical relevance statement:**

This review brings to light the commonly neglected/unknown factors negatively impacting contrast-medium injections and provides recommendations that can result in patient benefits, quality improvements, sustainability increases, and financial benefits by enabling otherwise unachievable optimization.

**Key Points:**

- How IV contrast media is administered is a rarely considered source of CT imaging variability.
- IV catheter selection, tubing length, injection systems, and insufficient flushing can result in unintended variability.
- These findings can be immediately addressed to improve standardization in contrast-enhanced CT imaging.

**Graphical Abstract:**

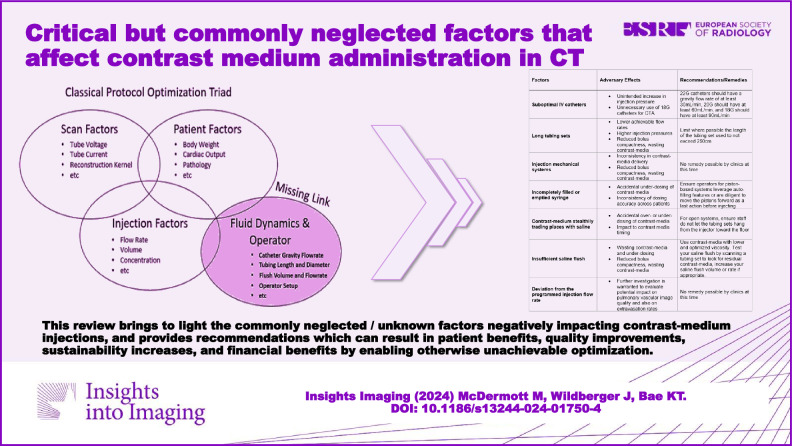

## Introduction

For decades, intravenous (IV) contrast media have been used to aid in diagnostic imaging of anatomical structures. In Computed Tomography (CT), the use of an iodinated contrast medium enables visualization of and differentiation between tissue types with similar densities, due to the x-ray absorption properties of iodine [1]. While the absorption characteristics are favorable, the increased viscosity of iodinated contrast medium (which increases non-linearly with increasing concentration) makes hand injection or the use of traditional infusion pumps infeasible at the clinically relevant flow rates and volumes required. Therefore, the use of power injectors to administer contrast media is standard practice in CT [2]. The standard setup includes an electromechanical pump, a protocol programming interface, a location to attach fluid sources, and plastic disposables (either syringes and/or tubing sets) designed to deliver the fluids from the source through the IV access device to the patient [3].

Since the late 1990s, when Bae et al published the pioneering work with a predictive computer model for contrast-medium enhancement in CT, the research and clinical focus on optimizing contrast-medium injection protocols accelerated [4–6]. Aided by a combination of advances in scanner technology, the concern over post-contrast acute kidney injury, growing utilization and indications of CT in diagnostic workups, and the recent sustainability questions (e.g., ground-water contamination by waste or excreted IV contrast-medium), the general direction of the field has been towards reducing the amount of injected contrast-medium wherever possible via optimized injection protocols.

In the last 2–3 decades, radiology practices have made significant strides in injection protocol optimization. Faster acquisition times from newer scanner models have enabled the reduction of contrast-medium doses through shorter injection durations [7–15]. In many clinics, weight-based dosing modifications have overtaken a single fixed protocol used for every patient. The clinical introduction of low-kilovolt imaging (down to 70 kVp from 120 kVp reference) depending on patient habitus and indication has further enabled the reduction in contrast-medium doses, in some cases by 50% or more from the reference protocols [16–19]. In addition, as reconstruction algorithms continue to advance, noise levels are improving. This paired with the use of virtual monoenergetic images obtained via dual-energy or spectral CT at lower kilo-electron-volt levels further reduces the amount of contrast medium needed [20]. This downward trend of administered contrast-media volumes, while beneficial for patients and clinics alike, comes at the expense of reducing the margin for error. There are little reserves for unexpected and unforeseen variation during data acquisition, and even small variations will have bigger negative effects on enhancement levels of the CT scan. It is therefore of utmost importance to look into the different parameters which are of major impact.

In this article, we review and discuss the current state, in particular, critical but commonly neglected factors affecting contrast-medium administration in CT. We leverage a combination of varying methodologies for uncovering and quantifying these confounding variables, including engineering benchtop equipment (e.g., Coriolis flow meters, pressure transducers, and volumetric measurement devices) combined with small, targeted systematic evaluations querying operators, equipment, and the physics and fluid dynamics at play. In the end, we propose how these critical factors should be considered and implemented in the contrast-medium administration protocols to optimize contrast enhancement. As authors, we bring robust expertise with more than 50 years of combined experience in research of contrast-medium injections and nearly 10 years of experience in the engineering development of injector systems.

### Factors determining contrast enhancement

**Fig. 1:**
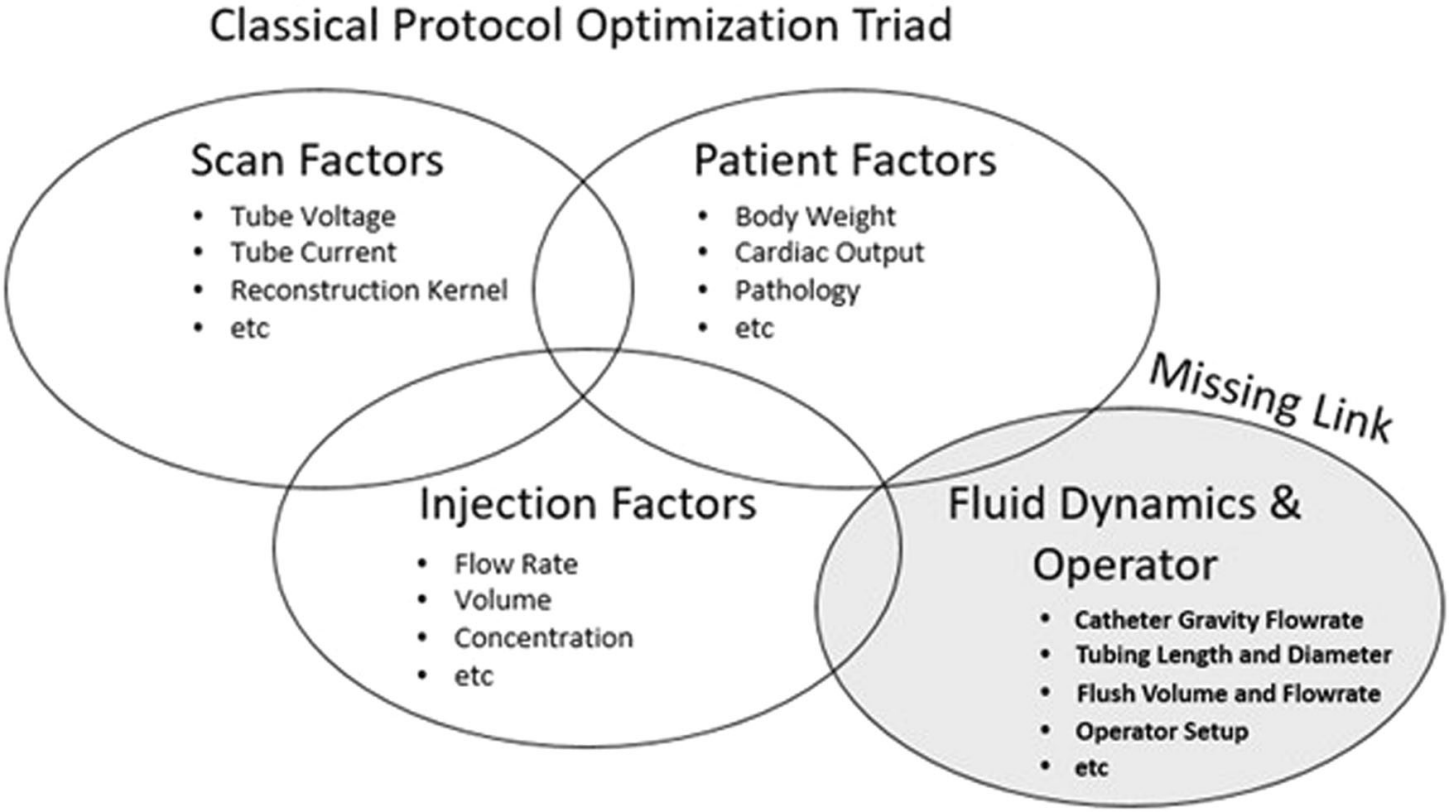
Injection protocol optimization triad and the missing category of error-inducing variables that have a significant impact on the outcome of a contrast-enhanced diagnostic procedure

Contrast-medium protocol optimizations were built on the foundational belief in a triad of relevant variables categorized by the early researchers: namely patient-related factors, CT-scan-related factors, and contrast-medium-injection-related factors. In this review, evidence is provided that a fourth bucket of crucial variables has been missed which account for previously unexplained phenomena and higher-than-expected variability in data (Fig. 1). While this new bucket does not have any direct impact on the factors of the other three, the factors from the well-known three buckets does directly affect the order or magnitude of the impact of the factors from the fourth bucket. This fourth bucket, if not understood and accounted for where possible, represents a barrier to further optimization of contrast-medium injection protocols.

## Selecting optimal IV catheters

The most common catheter gauge used in clinical practice is a 20-gauge (or 20 G), while 18 G may be used for higher flow rate applications, and 22 G or even 24 G may be used in more rare circumstances for small or difficult-to-access veins [21]. The gauge of the catheter represents the outside diameter of the device, with smaller numbers (e.g., 18 G) representing larger outer diameters. A common debate in clinics is which catheter gauge is appropriate for the desired flow rate. It is not uncommon for patients who come in with an existing IV access to have this switched to a bigger size to accommodate the higher flow rates needed for angiographic studies. This creates a certain amount of noncompliance and concern, especially among technicians operating the device and managing the patients. Fear of contrast-media extravasation drives much of this concern, and the tendency is toward larger catheter sizes for higher flow rate procedures even though this increases the likelihood of IV site pain and bruising for patients. This practice is fundamentally built on the belief that larger catheter sizes reduce injection pressure and enable higher flow rates.

**Fig. 2:**
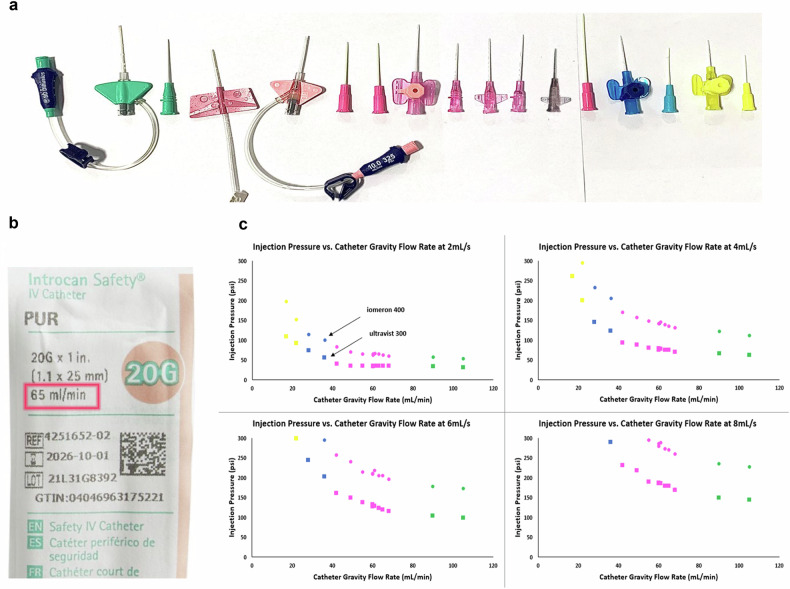
Critical factors to consider in the selection of IV catheter. **a** Various catheters color-coded according to their gauges, (**b**) an example of an IV catheter package with gravity flow rate highlighted, and (**c**) plots of injection pressure vs. catheter gravity flow rate for two different extremes of contrast-media (sub-datasets indicated with arrows) injected at 2, 4, 6, and 8 mL/s

While nearly all clinics are accustomed to looking at green, pink, blue, and yellow on the packages to select 18 G, 20 G, 22 G, and 24 G catheters (Fig. 2a), this is actually the incorrect number upon which to base the selection of IV catheters for power injection. The correct number, as proposed here, is also published on the package by nearly all IV catheter manufacturers—the Gravity Flow Rate (Fig. 2b). This is a standard measurement test that determines the flow rate at which water of a specific volume and head pressure will flow through the catheter under only the influence of gravity. This is an indirect measurement of the amount of pressure that the catheter will generate when fluid is injected through it, with higher gravity flow rates corresponding to lower pressures generated by injecting the same fluid at the same flow rate.

To demonstrate the correlation of this number, the authors evaluated the 16 different IV catheters each with a different gravity flow rate (2 × 18 G, 10 × 20 G, 2 × 22 G, and 2 × 24 G) across 5 different catheter manufacturers. A power injector was used to deliver two different contrast media through each of the different IV catheters at four different flow rates (2, 4, 6, and 8 mL/s). The two different contrast media used were 300 mgI/mL (iopromide, Bayer AG, Berlin, Germany) and 400 mgI/mL (Iomeron, Bracco Imaging, Milan, Italy) at body temperature representing the two clinically relevant extremes of contrast-medium concentrations (and subsequent viscosities) used in CT. Figure 2c shows the graphs of injection pressure vs. catheter gravity flow rate for each of the tested injection flow rates.

The data points are color coordinated by catheter gauge, and it is clearly shown that there is a strong correlation (minimum R^2^ = 0.9748) between injection pressure and IV catheter gravity flow rate with a decaying exponential relationship. This decaying exponential visually demonstrates the diminishing return at each flow rate of selecting any 18 G catheter over a high-performing 20 G. This is because the 20 G catheters with higher Gravity Flow Rates achieve roughly the same injection pressure as 18 G catheters (average difference of only 8 psi across all tested flow rates). In these cases, there is no additional clinical value in changing from the 20 G to the 18 G, and significant time, stress, and effort can be saved by the clinic.

In addition, as observed in Fig. 2 there is substantial variation in injection pressure within the same catheter gauges, especially among the 20 G (~ 60 psi). This highlights the importance of selecting an IV catheter with an optimized gravity flow rate, as ignoring this could result in unexpected performance degradation. This performance degradation includes CM injections aborted prematurely or delivered at a flow rate less than intended due to excessive pressure. This has the possibility of resulting in reduced image quality or non-diagnostic scans. This performance degradation can be mitigated by appropriate IV catheter selection. As demonstrated here, this selection should be based on the correct parameter that is relevant for performance: not catheter gauge and color but rather the gravity flow rate. As this becomes an economic-based purchasing decision, it is important to note that, to the best of the authors’ knowledge, the pricing and gravity flow rate are not causally linked for most manufacturers and therefore increasing performance likely will not be cost prohibitive. While a comparison of these results to previous studies would be ideal, an evaluation of 23,706 articles mentioning contrast-medium injections yields zero mentions of this parameter called Gravity Flow Rate for catheter selection.

Based on the data collected and experience, as a general rule, the authors recommend that 22 G catheters should have a gravity flow rate of at least 30 mL/min, 20 G should have at least 60 mL/min, and 18 G should have at least 95 mL/min. Selecting optimal IV catheters enables the minimization of injection pressure and maximization of achievable flow rates.

## Long tubing degrades injection rates and dosing accuracy

In many CT suites, space can be limited, and the positioning of an injector system can be challenging due to its size. For some procedures, also the patient may need to be positioned on the table through one side of the gantry that the injector system cannot reach. To avoid these spatial constraints, many clinics prefer to use longer patient lines than the standard 250 cm offered by many manufacturers. Further, some clinics may choose to attach additional tubing sets to the distal end of the tubing to increase flexibility (e.g., additional stopcock or valve) or to save costs by off-label multi-patient use of the longer tubing set.

In these cases, there are two negative implications on performance and dosing accuracy. The first is that the longer tubing length increases the pressure of the injection under the same conditions as shorter tubing sets. This is a basic fluid dynamics principle known as pressure drop. This is defined by the Hagen-Poiseuille equation, where the length of the tubing set is inversely proportional to the flow, however, under constant flow with a larger length, the pressure must increase correspondingly.
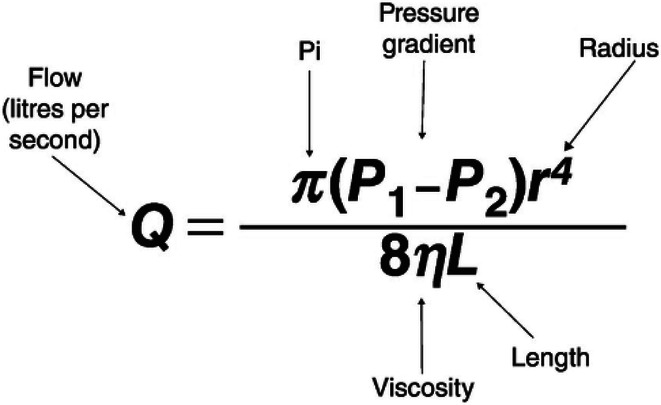


**Fig. 3:**
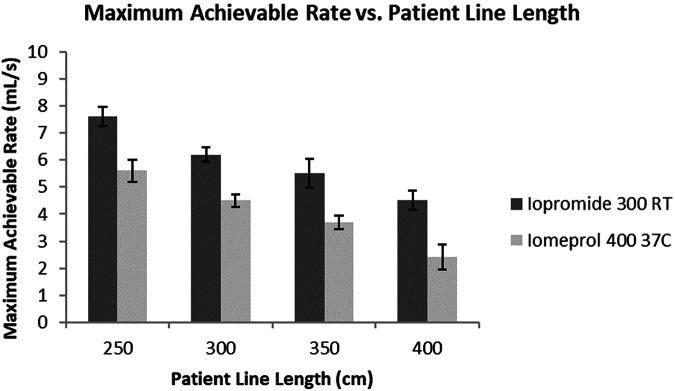
Plot of maximum achievable flow rate vs tubing length at two different conditions. The maximum achievable flow rate declines with an increase in the length of the tubing set obtained with two different contrast media at two different temperatures (RT, room temperature)

To demonstrate the impact of the longer patient lines on achievable flow rates, four different sizes were tested starting with the standard 250 cm and increasing every 50 cm up to 400 cm. Two different contrast media with different viscosities representing the clinically significant range of available contrast-media concentrations (300 mgI/mL and 400 mgI/mL) were injected up to a maximum rate before the injection system reached the pressure limit. In this case, the 300 mgI/mL was injected at room temperature and the 400 mgI/mL was injected at body temperature to reflect more common clinical practice. This maximum rate was recorded and compared across the different tubing set lengths as shown in Fig. 3. The increase of 150 cm from the standard length led to a corresponding decrease in achievable flow rates by 55% for the 400 mgI/mL and 40% for the 300 mgI/mL.

This significant decrease in achievable flow rates and the corresponding increase in pressure at constant flow rates has direct clinical implications. Reduction in achievable flow rates can limit the capability to deliver high iodine delivery rates necessary for angiographic studies, especially on larger patients. Also, the increase in injection pressure increases the negative effects on dosing accuracy, which are described in the following sections. While a comparison of these results to previous studies would be ideal, there is no existing literature that could be found that evaluates the impacts of tubing length on performance in CM injections.

It is recommended that every clinic evaluate any opportunity to position the injection system closer to the patient to reduce the need for the use of longer tubing sets. Although this may be impractical in some setups depending on the room design and the patient positioning within the scanner, attention should be paid to reducing the need for longer patient lines where possible.

## Injector mechanical systems affect contrast-medium delivery

The benefits of power injection systems in their ability to deliver higher flow rates and volumes in a more consistent manner than hand injections do not come without compromises. While it is obvious to view injection systems mainly along the lines of cost, features, accessories, and performance specifications (e.g., maximum flow rates and maximum pressure limits), the more important aspect when it comes to performance and the contribution to consistent image quality are the plastic disposables material and geometry combined with the mechanism of delivery. A number of injectors available in different brands and classifications on the market fall into one of three categories: piston-based, peristaltic pump, or hydraulic. Piston-based injectors load fluid into syringes or reservoirs which are then expelled through tubing sets into the patient by a piston/plunger (otherwise thought of as an automated hand syringe). Peristaltic pumps use rotational motion to pinch and un-pinch sections of a tube which draw fluid from a supply and inject it into the patient through an additional tubing set. Hydraulic injectors use fluid external pressure on a collapsible reservoir to compress the reservoir at a controlled rate and expel the fluid into the tubing sets to be delivered to the patient.

**Fig. 4:**
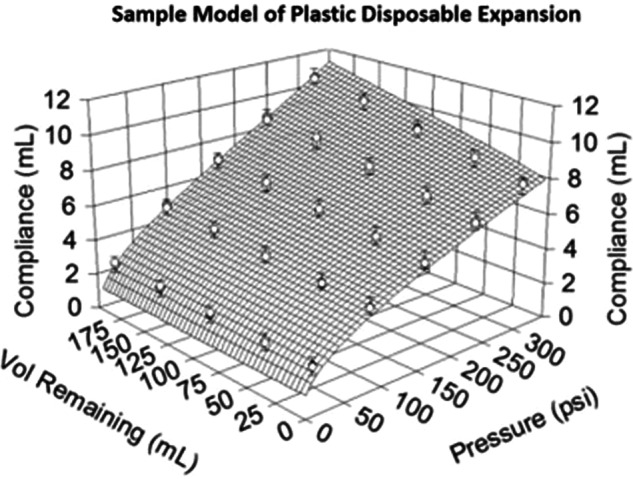
Mathematical simulation illustrating the effect of expanding plastic disposables. “Compliance” is affected by both the tubing set volume and pressure.

A problem common to all manufacturers regardless of delivery mechanism, is that the plastic disposables swell and stretch under the internal loads of the high injection pressures. This swelling and stretching is dependent upon temperature, injection pressure, reservoir volume or tubing volume, and material properties of the plastic. The loads at high injection pressures can be extreme for thin and flexible plastics to endure; for context, 300 psi of pressure in an example syringe can equate to 800 lbs or 363 kg of force pressing on every surface of the plastic. Figure 4 illustrates a mathematical simulation generated in this study of the non-linear expansion of the plastic disposables from an example piston-based injection system. In this simulation, the plastic disposables under high injection pressure can expand up to 10 mL in extra internal volume. The worst case as measured by the authors is on a hydraulic injection system with nearly 50 mL of additional volume swelling. By Conservation Law, this means that during an injection, while the expectation is that fluid is being delivered to the patient, the reality is that much of the volume is actually being injected into the expanding plastic disposables. This creates an unexpected delay in delivery of the bolus which is non-linear and dependent upon injection pressure. As the plastic disposables are elastically deforming under pressure but have not yielded into a new shape, when the pressure is relieved at the end of the injection the disposables decompress, expelling the extra trapped volume into the patient at a decaying rate.

**Fig. 5:**
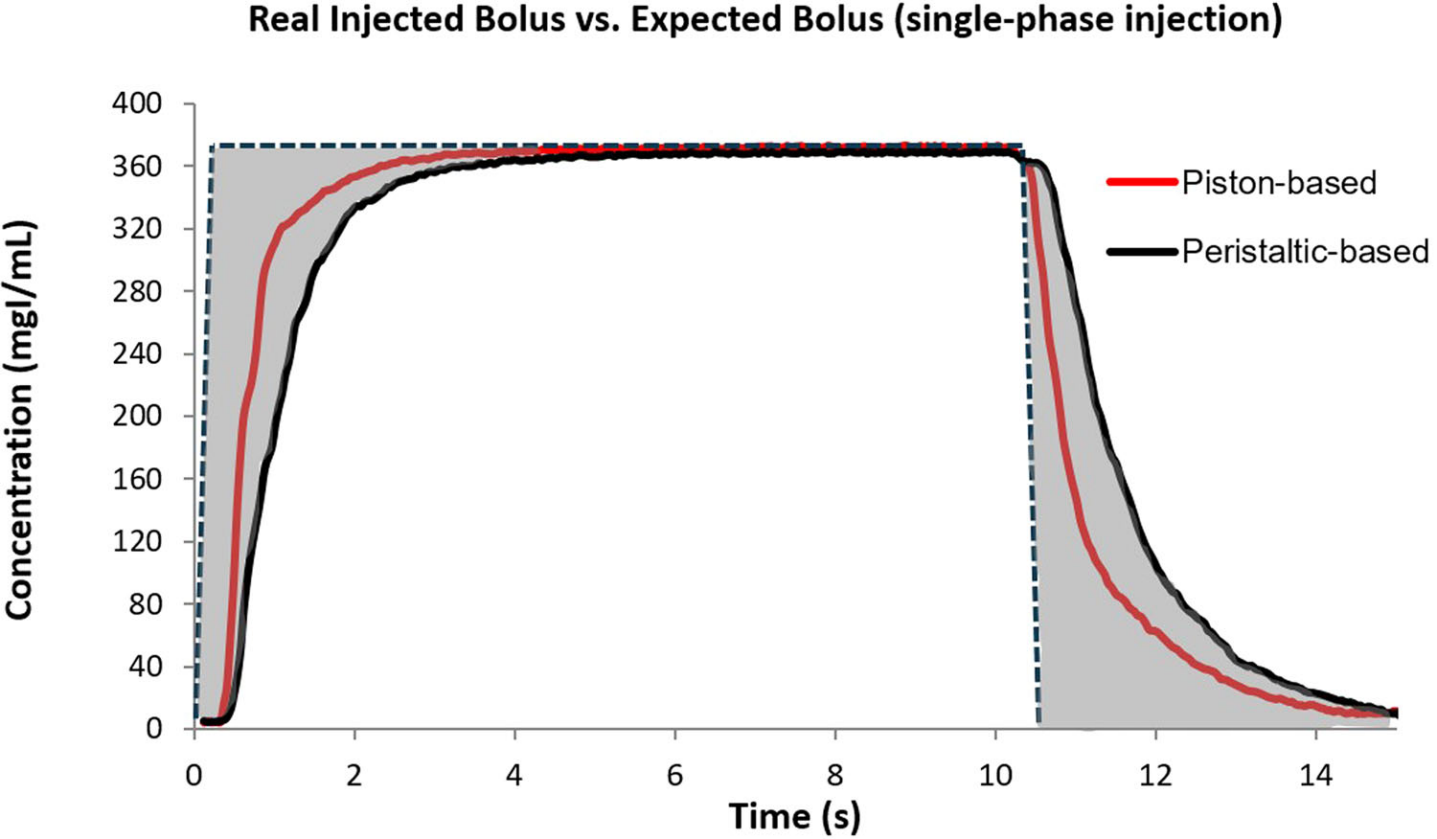
Concentration-time plot of ideal versus empirical injected flow rates for a single-phase injection. The shaded area represents the deviation between the ideal (dotted line) and empirical (solid curves measured from two different systems) injected flow rates as caused by confounding factors like plastic disposables expansion

The clinical implications of this factor are two-fold. First, the trapped volume from the expanding disposables is not a contributor to the main bolus of the injection, as this volume is only released when the injection pressure is relieved (partially during the saline flush and the remaining volume at the end of the injection). Therefore, the desired iodine delivery rate is not achieved for the intended duration, and the total effective iodine load (contributing to parenchymal enhancement) administered is likely less than intended. This phenomenon was measured using a real-time Coriolis density and flow meter (consistent with previous literature for assessing injected iodine concentrations), with the results from an example injection of CM with an iodine concentration of 370 mgI/mL (with a viscosity representing the middle of the range of available contrast-medium) at 4 mL/s for 40 mL as shown in Fig. 5. This was repeated with a piston-based injector and a peristaltic injector to simulate the two configurations with the most significantly different disposables designs. This figure displays the injected concentration over time entering the patient’s circulation. The magnitude of the shaded region at the beginning of the injection is a direct correlation to the expansion of the plastic disposables. The shape of the shaded region at the end of the injection is a combination of decompression of the plastic disposables as well as the efficacy of the saline flush in eliminating the contrast medium remaining in the tube. This latter contributor will be discussed in more detail below.

The second clinical implication is that the enhancement achieved in a target body region will vary significantly even with the exact same injection protocol for the exact same patient as long as any variable is changed that affects injection pressure (and therefore disposables expansion) [4–6]. These variables include contrast-media viscosity, length and diameter of the tubing sets used, size/type of IV access device, etc. This holds true even if the exact same injection system itself is used. When adding in the different types of injection systems, the inconsistency may be even more substantial.

**Fig. 6:**
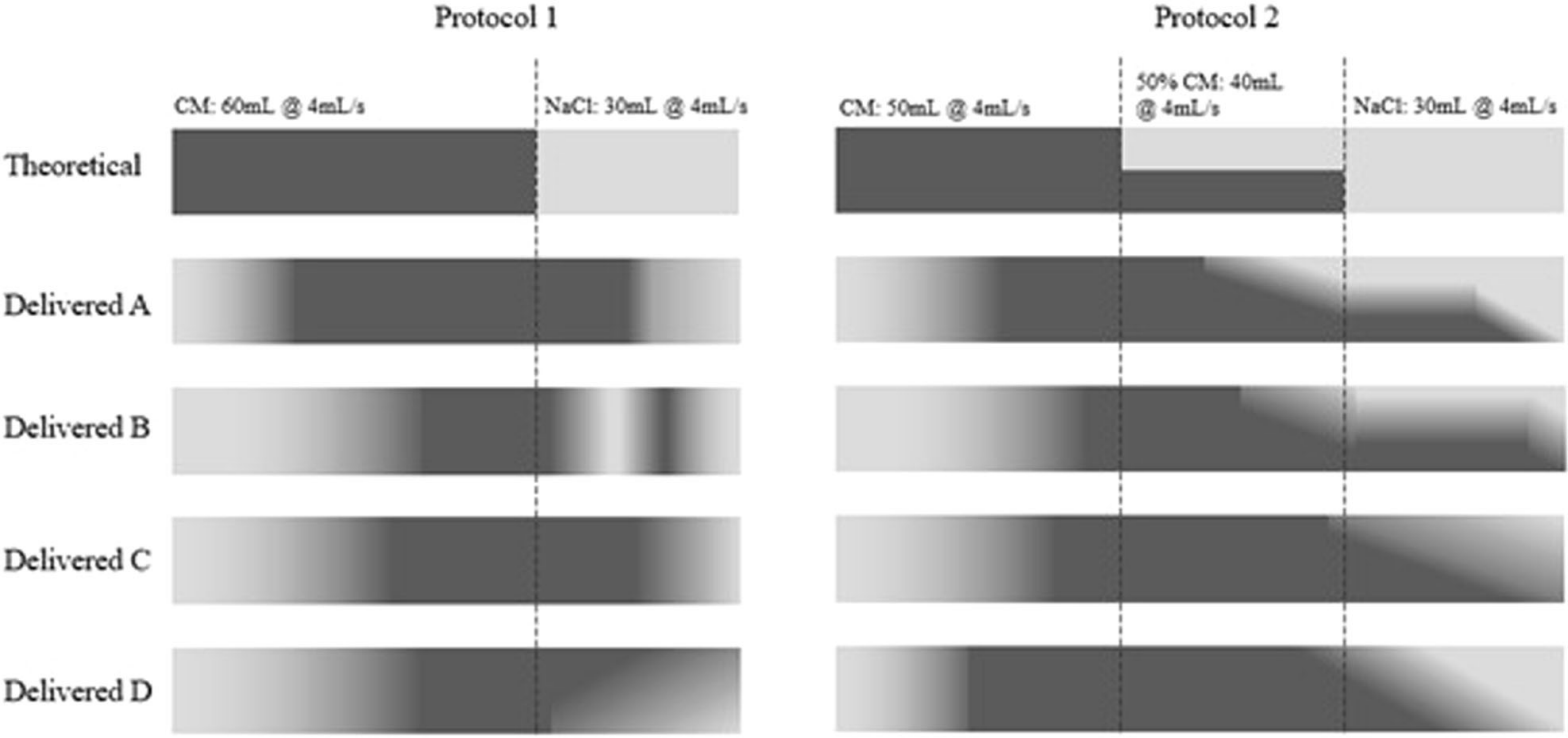
Contrast-medium and saline distribution profiles at two different injection protocols delivered on four different injection systems (two piston-based, two peristaltic pumps). The two injection protocols are shown in the header of the figure. The greyscale shading over time correlates with the injected concentration of the fluid as measured just proximal to the IV access device for each setup

Figure 6 shows the contrast-medium and saline distribution profiles at two different injection protocols delivered across four different clinical setups with varying injection systems, contrast media viscosity, and IV catheter. The greyscale shading over time is directly correlated to the injected concentration of the fluid (equivalent method as described above). Each of the four different setups used a different injection system (two piston-based, two peristaltic-pump) configured each with a contrast-medium of constant concentration (370 mgI/mL) but different viscosity (5 cP, 12 cP, 17 cP, 22 cP), and each with a different IV catheter gauge and gravity flow rate (18 G 105 mL/min, 20 G 65 mL/min, 20 G 42 mL/min, 22 G 36 mL/min).

The general expectation from clinicians would be that the delivered output of the CM to the patient would be roughly identical. As shown in the figure, even with an identical contrast-medium injection protocol, the output as delivered by the different setups is significantly altered from the expectation. Note that these variations are measured at the catheter site prior to the patient and represent variability in the input function. The variability introduced by patient-related factors has not yet even been added to the equation at this stage.

Because of the variable contrast-medium delivery caused by the plastic disposables expansion and injector mechanism, the seemingly same injection protocol would yield significantly different contrast enhancement. Unfortunately, there is no easy fix for this that can be attempted from the side of practicing clinicians. To compensate for this, the manufacturers of the disposables would need to significantly increase the strength of the materials used which adds substantial cost that would be economically unviable. The other alternative is to use mathematical modeling to predict the expansion of the disposables under given conditions and to compensate for this in real-time. In whichever way this previously undiscussed phenomenon is to be addressed, there is no clear-cut solution for everyday clinical practice at the moment.

## Improper injector setup causes under-dosing

**Fig. 7:**
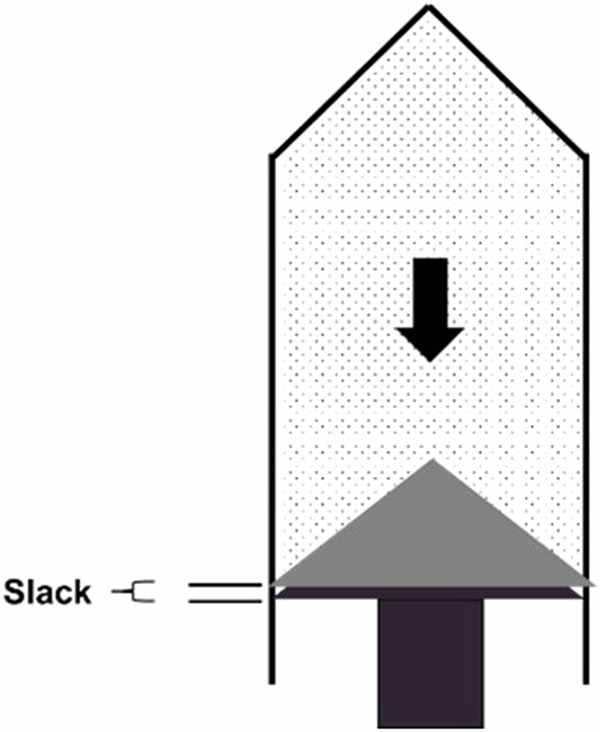
Example diagram showing where slack can be observed in a typical piston-based injection system

When using a piston-based injector system with syringes, benefits have been shown including the ability to deliver more consistent flow than peristaltic pump-based systems as well as higher achievable iodine delivery rates [3, 13]. These benefits do not come without tradeoffs, which can easily be compensated for if they are understood. The key operating step of some piston-based systems that leads to the most common operator error is on filling of the syringes or reservoirs. For systems that allow the user to manually fill the syringes by retracting the piston, at the end of the filling process, if the plunger is not manually driven forward far enough, the system may under-deliver the corresponding fluid. This is due to the fact that all mechanical systems have slack in them. When the plunger is driven backward to fill the syringes, the many different mechanical components pull against each other and compress. This creates small gaps between the different components. If the plunger is not then correspondingly driven forward a sufficient amount to close these gaps, when the injection starts there will be a motion of the piston forward that does not result in displacement of the fluid out of the syringe and into the patient. This is shown in Fig. 7. On many syringe-based systems, every 0.5–0.75 mm of linear motion corresponds to 1 mL of fluid. Therefore every 0.5–0.75 mm of mechanical slack that is not accounted for will result in 1 mL of under-delivered volume vs. the programmed protocol. This is known by manufacturers and has been accounted for with automated filling features, however, many operators still use the manual filling options.

A small observational study of 20 patient procedures performed by technical staff in the author’s clinic yielded an average under-delivered volume of 3.6 mL ± 3.2 mL per injection with a maximum of 12 mL. After discussion with the technical staff about the unintended error and a small change to the operating procedure to either use automatic filling features or to ensure that the plunger has been pushed forward after manual filling, the average under-delivered volume decreased to 0.0 mL with no observable cases of the error in the study.

Although this may be a smaller magnitude, an average of 3.6 mL in contemporary procedures may represent 5–10% of the total injected contrast-medium volume depending on the indication. A maximum of 12 mL represents an even more significant portion of the intended contrast-medium volume injected. This previously unknown error was likely accounted for in the empirical determination of the site-specific contrast injection protocol, and variation is likely masked by blaming image quality implications on patient-related factors or scan timing. This is the first evaluation of its kind in literature to the best of the author's knowledge, therefore comparison to existing literature is unfortunately not possible.

It is recommended that radiographer/technician staff evaluate the variability in the setup of the injection systems among their teams and agree upon a standard operating procedure. This should be checked using water and a weighing scale or a simple graduated cylinder to ensure the volumetric accuracy of the first injection after setup.

## Contrast-medium stealthily trades places with saline

**Fig. 8:**
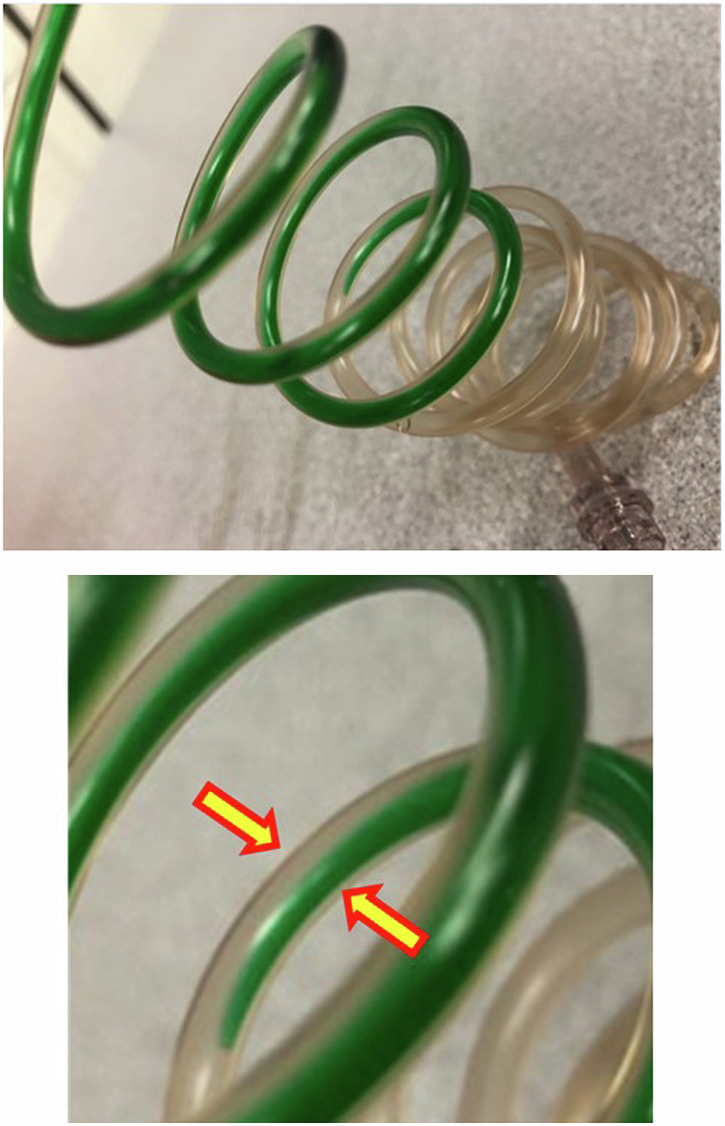
A tubing set showing contrast-medium trading places with saline in (left) a large field of view and (right) a magnified view at the interface of the contrast-medium and saline. The contrast medium (dyed in green) filled in the reservoirs migrates downward to trade places with saline as the density of the contrast medium is greater. The magnified view with arrows reveals that the two fluids layer and slide past each other with no other driving force besides gravity

Iodinated contrast media and saline solution (NaCl) are significantly different in both viscosity and density. The density of iodinated contrast medium is typically between 1.3 and 1.45 g/cm^3^, while the density of saline is approximately 1.0 g/cm^3^. In normal clinical practice (excluding pediatric patients and neonates where minimizing injected volume is critical), NaCl solution is typically used to prime the tubing sets prior to injection. The volume of these tubing sets can be between 5 and 26 mL in volume depending on the manufacturer and length. The tubing sets are typically positioned such that the fluid source at the level of the operator is higher than the end of the tubing set which is closer to the ground. Because of their difference in density, the heavier contrast that is either in the fluid supply or in the syringes will exchange places with the saline in the tubing set. In particular, the impacts of density and gravity immediately take effect in an “open system”: that is, in piston-based systems where there are no stopcocks or check valves attached to the syringes, or in peristaltic-pump systems when the different fluid supply lines are intermittently open to each other as pinch valves change position, Fig. 8 shows an image of the layering of the fluids as the contrast-medium is dyed green for visualization. The saline that was used to prime the tubing sets will flow upward into the contrast-medium supply and the contrast-medium will instead fill the tubing set. An average-sized tubing set that is 10 mL in volume has been observed to trade places in less than 30 s under normal conditions.

The clinical implication is that 10 mL of contrast medium is delivered more than intended, as the saline from the tubing set floats to the top of the contrast supply. The secondary consequence of this is that over the course of the day, the contrast-medium supply will become gradually more and more diluted. Therefore, the end effect is that early patients when the contrast-medium supply is first filled will receive an unexpectedly high dose. The last patient for that contrast-medium supply will receive an unexpectedly low dose as it has been diluted over time. Based on the known tubing set volumes from available manufacturers, the magnitude of this over-delivery in a worst-case scenario can reach 26 mL. Last, if the contrast-media bolus starts at the end of the tubing, instead of the beginning, the timing of the arrival of the contrast-media will be different from expectations and could affect image quality. To the best of the author's knowledge, this is the first mention of this phenomenon in the literature.

It is recommended that clinics ensure their staff are not leaving the tubing sets (when open systems are in use) in a position where they hang lower than the fluid supplies on the injector system.

## Insufficient saline flush wastes contrast media

Several studies have been conducted evaluating the performance of a saline chaser, which was predominantly introduced into clinical practice to flush the otherwise wasted contrast medium from the tubing set and the peripheral veins of the patient into systemic circulation [22, 23]. Evidence also suggests that a saline chaser keeps the contrast bolus more compact and enables higher peak attenuation and less bolus dilution through the pulmonary circulation and the capillary effects of the lungs [24].

**Fig. 9:**
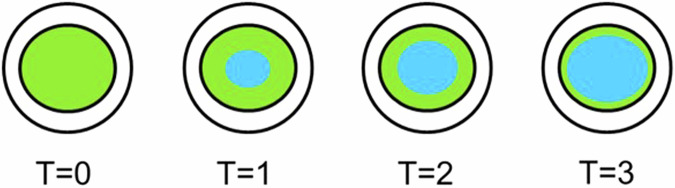
Boundary layer phenomena between saline and contrast-medium in a tubing cross-section at four time points. The central blue region represents saline chaser, while the peripheral green region represents contrast-medium. Insufficient saline chasers would fail to completely clear the residual layers of contrast-medium

When we determine an adequate amount of saline chase, there are important considerations to keep in mind in view of the injection fluid dynamics which is influenced by the differences in the viscosity and density between contrast-medium and saline. The saline chaser does not push the contrast-medium forward like solid objects, i.e., plug flow. Rather, the thin fluid of saline chaser mixes and shears through the thick center layers of contrast-medium, thereby leaving a boundary layer of contrast-medium behind stuck to the walls of the tubing set. The larger the chaser volume at a constant flow rate, the less contrast medium is left behind in the tubing set. Also, the higher the saline chaser flow rate, the more turbulent the flow becomes, and the less contrast medium is left behind in the tubing set at the same chaser volume with a lower flow rate. Figure 9 demonstrates the fluid dynamics phenomena of the boundary layer at three different time points after the initiation of the saline flush.

**Fig. 10:**
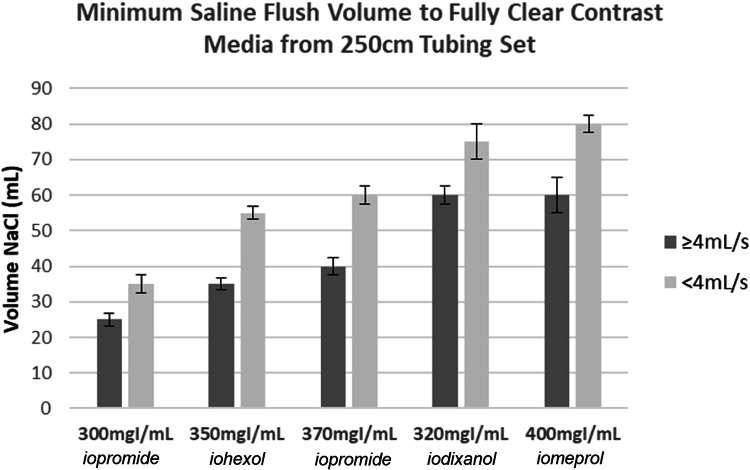
Plot of the minimum saline flush volume required to clear contrast media of different concentrations at flow rates below 4 mL/s or at and above 4 mL/s. A higher volume of saline flush is required to clear contrast media of higher concentration injected at a lower rate

To quantify this phenomenon, an experimental study was conducted using contrast media of varying viscosities and concentrations (300 mgI/mL, 320 mgI/mL, 350 mgI/mL, 370 mgI/mL, and 400 mgI/mL). The contrast media were filled into a standard 250 cm tubing set and saline flushes of varying volumes and flow rates were pushed through the tube with a power injector. The same Coriolis meter previously discussed was used to measure the concentration of the fluid exiting the tubing set. A flush was considered 100% successful when the density/concentration of the fluid exiting the tubing set reached that of the 0.9% NaCl solution used as the flush (each test was repeated three times enabling standard deviations to be calculated). Figure 10 shows the compiled results across all tested contrast-medium concentrations with averages for flow rates below 4 mL/s and at or above 4 mL/s. A threshold of 4 mL/s was selected as this is the transition point from laminar to turbulent flow for NaCl solution in a standard tubing set, which significantly affects the capability to flush contrast media from the boundary layer. This is evidenced in the figure below with significant differences in minimum flush volume above and below this flow rate threshold.

Comparing these results with the usual clinical practice where saline flush volumes are between 20 and 40 mL on average and flow rates outside of CTA procedures are typically below 4 mL/s, it is expected that there is significant wasted contrast media unknowingly being discarded within the tubing sets. When evaluating contrast-medium volumes left in the tubing set after 30 mL of a saline flush, the average volume wasted was 1.4 mL with a minimum of 0.3 mL and a maximum of 3.8 mL. This was measured by differences in weight measured on a highly accurate scale and calculating the volume based on measured contrast-medium density. In the clinic of the authors, this corresponds to a waste of 22.5 liters of contrast media each year. This drives concern from both an economical and an environmental perspective. The higher the concentration/viscosity of the contrast medium, the increase in the wasted contrast medium thrown away after the saline flush. Further, as discussed above, the increase in the length of the tubing set compounds this issue further and increases the minimum volume of flush needed to successfully remove all contrast media from the tubing.

An additional element of concern is performance degradation. The less effective the saline flush is at removing the contrast media from the tubing, the further separated that residual contrast media becomes from the main bolus that has already entered the circulatory system of the patient. In dynamic studies where iodine delivery rate and bolus compactness are critical metrics contributing to attenuation, insufficient saline flush results in larger volumes of contrast media intended to be part of the main bolus that instead lag too far behind to provide diagnostic relevance, or never enter the patient at all and is discarded in the tubing set.

It is recommended that clinics evaluate their current saline flush volumes as part of the set injection protocols. A simple mechanism to observe whether contrast-medium is left behind in the tubing set is to put the used tubing in the scanner prior to discarding and to scan at 70–90 kVp. Any contrast medium present in the tubing set will be visible and suggest that the flush volume is insufficient. To the best of the authors’ knowledge, while saline flush volumes have been sporadically evaluated in the literature, the efficacy of the saline flush to clear the contrast media has not been previously investigated.

## Deviation from the programmed injection flow rate

**Fig. 11:**
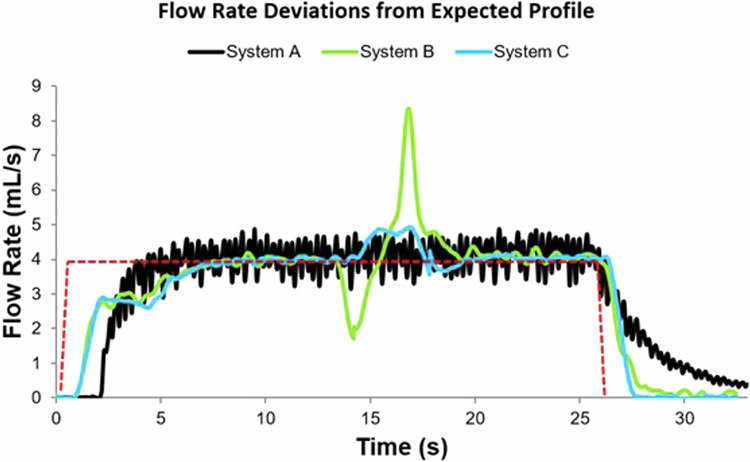
Flow rate fluctuations from the ideal profile in three different by injection systems. **a** Flow rate fluctuations with a peristaltic-based injection (black curve). **b** Flow rate fluctuations with a piston-based injection with a transitional drop in flow rate at the switch of contrast-medium to saline (green curve). **c** Flow rate fluctuations with a piston-based injection with lower effect of plastic disposables compliance (blue curve).

The widely unknown physical phenomena mentioned above like plastic disposables expansion and fluid property differences of contrast-medium and saline also contribute to unexpected variations in flow rate. Power injection systems are believed to deliver consistent flow rates and volumes at high pressures beyond what is possible from human hand injection. However, from the information provided by the injector manufacturers on the user interfaces, it is not possible to audit whether this flow rate and delivered volume are truly consistent. As seen from the examples above, there are many cases where the expected delivered volume can vary greatly from reality, however, no indication of this is provided. The same is true with flow rates. For example, peristaltic injectors deliver fluid by roller pumps constantly pinching and un-pinching tubing to drive fluid forward. This action results in sinusoidal flow rate variation that significantly deviates from expected, as was proven by Chaya et al [25]. A representation of these findings also confirmed in this study is shown in Fig. 11.

While this flow rate variation occurs on peristaltic systems, unexpected variations in flow rates also occur on piston-based and hydraulic systems, albeit a lesser magnitude. At the transition between contrast-medium and saline flush, there is a significant momentum change when the lower viscosity fluid reaches the catheter. This results in a drop in pressure that is visible on the pressure graph on the injector display. However, when the pressure decreases rapidly, the plastic disposables also decompress, with this decompression forcing additional fluid out of the system. This causes a momentary variation in flow rate that can deviate significantly from what is expected (Fig. 11), This phenomenon is made worse with higher viscosity contrast-medium, as the change in pressure is significantly higher. Although these fluctuations in flow rate likely have little clinical impact on image quality as the deviation is smoothed out by the capacitive effect of the lungs (note: dynamic imaging of the pulmonary arterial vessels may be impacted), further studies are warranted to investigate the effect of the flow rate deviations caused by injector systems on IV catheter displacement and subsequent extravasation.

## Summary and recommendations

As shown here in this paper, there are many different factors associated with the contrast-medium injection that are not widely known or understood, from equipment selection to operator variability and also fluid dynamics. Although these factors can contribute to significant error and variability in the quality and consistency of contrast-medium delivery, they were rarely investigated or addressed in the design and implementation of injection protocols. When contrast enhancement is unexpectedly suboptimal, we often blame intrinsic patient factors or are just puzzled without knowing what to do. Understanding these critical but commonly neglected technical factors is an opportunity for wider awareness and correction where possible. There are two main reasons for this. The first is that as technology continues to advance, contrast-medium volumes per procedure continue to be driven lower. With lower procedure volumes, the sources of error that in the past were single-digit percent errors and easily missed will become substantial sources of error within the procedure. Wasted contrast-media volumes that could otherwise be optimized will have a greater effect on image quality consistency. This waste drives not just quality challenges but also unnecessary costs and negative impacts on sustainability. In a time where contrast-medium shortages drive significant stress within hospital systems, responsible use of contrast-medium is highly recommended.

There are simple steps that can be taken to address many of the sources of error/waste identified in the paper (Table 1). The authors recommend evaluation of the gravity flow rate of the catheters in use, switching to the use of shorter tubing sets where appropriate, and assessment of the quality and consistency in the value chain of contrast-enhanced exams; from the injection system and contrast-media to the operators and handling/setup procedures in clinical practice. Elimination of these hidden sources of error can bring immediate benefits while paving the way for the future where predictive modeling as introduced by Bae et al decades ago can be reasonably implemented in the clinic to drive standardized and fully optimized contrast-media injections that have so far eluded us.

**Table 1:** Summary of factors, adversary effects, and recommendations

Factors	Adversary effects	Recommendations/remedies
Suboptimal IV catheters	• Unintended increase in injection pressure • Unnecessary use of 18 G catheters for CTA	22 G catheters should have a gravity flow rate of at least 30 mL/min, 20 G should have at least 60 mL/min, and 18 G should have at least 90 mL/min
Long tubing sets	• Lower achievable flow rates • Higher injection pressures • Reduced bolus compactness, wasting contrast-media	Limit where possible the length of the tubing set used to not exceed 250 cm
Injection mechanical systems	• Inconsistency in contrast-media delivery • Reduced bolus compactness, wasting contrast-media	No remedy is possible by clinics at this time
Incompletely filled or emptied syringe	• Accidental under-dosing of contrast-media • Inconsistency of dosing accuracy across patients	Ensure operators for piston-based systems leverage auto-filling features or are diligent in moving the pistons forward as a last action before injecting
Contrast-medium stealthily trading places with saline	• Accidental over- or under-dosing of contrast-media • Impact to contrast media timing	For open systems, ensure staff do not let the tubing sets hang from the injector toward the floor
Insufficient saline flush	• Wasting contrast-media and under-dosing • Reduced bolus compactness, wasting contrast-media	Use contrast media with lower and optimized viscosity. Test your saline flush by scanning a tubing set to look for residual contrast-media, increase your saline flush volume or rate if appropriate.
Deviation from the programmed injection flow rate	• Further investigation is warranted to evaluate the potential impact on pulmonary vascular image quality and also on extravasation rates	No remedy is possible by clinics at this time

*CTA* CT coronary angiography, *G* gauge, *mL/min* milliliters per minute

## Data Availability

All data generated in the context of this article are freely available upon request made to the corresponding author.

## Abbreviations

IV: Intravenous
kVp: Peak kilovolt
NaCl: Saline solution
